# Responsiveness of respiratory function in Parkinson’s Disease to an integrative exercise programme: A prospective cohort study

**DOI:** 10.1371/journal.pone.0301433

**Published:** 2024-03-29

**Authors:** Laura McMahon, Denise McGrath, Catherine Blake, Olive Lennon

**Affiliations:** Health Sciences Centre, UCD School of Public Health, Physiotherapy and Population Science, University College Dublin, Dublin, Ireland; University of Sharjah, UNITED ARAB EMIRATES

## Abstract

**Introduction:**

Respiratory disorders are the most common cause of death in Parkinson’s Disease (PD). Conflicting data exist on the aetiology of respiratory dysfunction in PD and few studies examine the effects of exercise-based interventions on respiratory measures. This study was conducted to better understand respiratory dysfunction in PD and to identify measures of dysfunction responsive to an integrative exercise programme.

**Objectives:**

The objectives were to compare baseline respiratory measures with matched, published population norms and to examine immediate and longer-term effects of a 12-week integrated exercise programme on these measures.

**Design:**

Twenty-three people with mild PD (median Hoehn & Yahr = 2) self-selected to participate in this exploratory prospective cohort study. Evaluation of participants occurred at three time points: at baseline; following the 12-week exercise programme and at 4-month follow-up.

**Outcome measures:**

Outcome measures included: Forced Vital Capacity (FVC), Forced Expiratory Volume in 1 second (FEV1), FEV1/FVC ratio, Peak Expiratory Flow (PEF), Inspiratory Muscle Strength (MIP), Expiratory Muscle Strength (MEP), Peak Cough Flow (PCF), and Cardiovascular Fitness measures of estimated VO2 max and 6-Minute Walk Test (6MWT).

**Results:**

Compared to published norms, participants had impaired cough, reduced respiratory muscle strength, FEV, FVC, PEF and cardiovascular fitness. Post exercise intervention, statistically significant improvements were noted in MEP, cardiovascular fitness, and PEF. However only gains in PEF were maintained at 4-month follow-up.

**Conclusions:**

Significant respiratory dysfunction exists, even in the early stages of PD. Metrics of respiratory muscle strength, peak expiratory flow and cardiovascular fitness appear responsive to an integrative exercise programme.

## Introduction

Impairments in Parkinson’s Disease (PD) include respiratory dysfunction, noted even in early disease stages and asymptomatic patients [1]. Respiratory disorders including pneumonias are the most common cause of death in PD [2] and are reported to cause diminished quality of life [3]. While aspiration pneumonias secondary to dysphagia are typical in the advanced disease stages [4], a review of hospital admissions for individuals with PD, found 32.5% of admissions were as a result of respiratory system diseases, excluding aspiration pneumonias [5]. This finding is replicated in other European research which noted increased hospital mortality, length of stay and health care costs associated with respiratory system diseases in PD [6, 7].

Controversy and conflicting data exist in the literature about the aetiology of respiratory dysfunction in PD [8–12]. A narrative review highlights restrictive, obstructive, central ventilatory control and drug related side effects (e.g., levodopa-induced diaphragmatic dyskinesis) as the primary causes. Mechanisms of restrictive pulmonary disease in PD include myopathy, autonomic dysregulation, and rigidity/bradykinesia. Direct basal ganglia involvement and axial dystonia are presented as potential mechanisms in obstructive disease. Mechanisms for central dysfunction include direct brainstem involvement and indirect ST-PAG-RTN pathway hypofunction. Drug related side effects (intake and withdrawal), include pulmonary fibrosis, dyskinesias and neuroleptic-like malignant syndrome [13]. A meta-analysis of respiratory measures in PD in comparison to matched controls identifies a predominantly restrictive pattern of dysfunction with reduced lung volumes, decreased inspiratory muscle strength and an ineffective cough mechanism [14]. Both reviews conclude the growing need to test for pulmonary dysfunction and identify appropriate preventative and management strategies in PD [12, 13].

The benefits of exercise-based interventions on motor impairments in PD are well established for outcomes of strength and cardiorespiratory fitness [15–17], motor components of the UPDRS [18], gait [19, 20] and balance [17, 20, 21]. Interventions commonly used include Resistance Training (RT), Endurance Training (ET) and Other Intensive Training Modalities [17] that vary from boxing, dance, tai-chi to yoga. By comparison, a relatively small body of literature examines the effects of exercise-based interventions on respiratory function in PD. Much of the existing literature in this area directly targets the respiratory muscles using approaches such as incentive spirometry to improve lung volumes [22] or respiratory muscle strength training to improve inspiratory muscle strength [23–25]. A systematic review of non-pharmacological interventions to improve respiratory function in PD concluded that varied forms of exercise can affect change in respiratory outcomes [26]. Pooled data from studies examining RT, ET and functional task training demonstrated significant effects for outcomes of inspiratory muscle strength, expiratory muscle strength and peak expiratory flow (PEF) rate.

Given the multifaceted potential benefits of exercise for managing PD, community-based group exercise programmes are becoming more prevalent to support greater numbers of people beyond the medical setting. This pilot study tests an integrative exercise regime comprising three different types of opt-in exercise classes to improve both mobility and respiratory outcomes in people with PD. The opt-in classes were high intensity interval training in the form of spin classes; Tai Chi designed for people with chronic disease and a functional circuit training class catering to participants with different levels of mobility. Our study was carried out in tandem with a feasibility study exploring the acceptability, scalability, safety, and sustainability of the university-based, integrative exercise programme for a cohort of community-dwelling people with PD [27].

To date, there are no published data available on respiratory dysfunction in people with PD presenting to a community-based exercise programme. Given the availability of general exercise programmes, it is important to establish if these programmes affect respiratory function in people with PD. Therefore, this nested study was conducted to better understand common respiratory dysfunction in people with PD presenting to a community-based exercise programme and to identify what measures of respiratory function are potentially responsive to the integrative exercise programme.

## Materials & methods

### Study design

An exploratory prospective cohort study. Data collection took place in a laboratory setting and the exercise programme was delivered as part of a community initiative delivered at a university gym setting.

### Participants

Members of a local PD organisation were invited to participate in the exercise programme at the university. An information leaflet was provided to all potential participants. All study participants, volunteers by self-selection, provided written informed consent prior to participation. Evaluation of participants occurred at three time points during the study, an initial baseline assessment, following completion of the 12-week programme and at 16-weeks post programme completion. Ethical approval for the study was provided by the Human Research Ethics Committee at the University (Ref: xx-xx-xx).

### Pre-screening

Pre-Screening was a 3-phase process. Potential participants were first screened by phone for study suitability against the following inclusion criteria; confirmed diagnosis of Parkinson’s Disease, under the care of a consultant neurologist, mobile, of sufficient cognitive status to give informed consent (later confirmed with the Mini-mental State Exam) and sufficient fluency in English to understand the information and consent sheet; and exclusion criteria: chronic respiratory disease diagnosis (e.g., COPD), acute respiratory tract infection, unstable medical conditions (e.g. uncontrolled diabetes), and dementia. Screening for suitability to participate in an exercise programme was conducted by phone using the Canadian Society for Exercise Physiology “Get Active Questionnaire”. If deemed suitable, participants were invited to the laboratory for comprehensive screening. Vitals including resting heart rate, blood pressure, respiratory rate and oxygen saturation levels were recorded. Where vitals were outside the safe limits for exercise participation as defined by the Canadian Society for Exercise Physiology, (resting HR >100 bpm or systolic BP >160 mmHg or diastolic BP >90 mmHg) participants were excluded and referred to their general practitioner. The third level of screening included a six-minute submaximal graded cycle ergometer test (YMCA protocol) to estimate VO2 max. Heart rate and blood pressure responses to exercise were monitored over the test and for four minutes afterwards to screen for autonomic dysfunction manifesting as exercise-induced hypotension. Those with abnormal cardiovascular responses to exercise were excluded from the high intensity aerobic spinning classes.

### Assessment protocol

Basic demographic details and history of PD and respiratory illnesses were recorded using a proforma. Anthropometric measurements were taken. Participants were assessed using the MDS-UPDRS [28] and total and subscale scores calculated [29]. Medication on or off phase was recorded for those on levodopa medication. Respiratory rate was calculated with participants sitting in a chair, the number of breaths in a 60 second period was recorded while oxygen saturation was being recorded.

#### Spirometry assessment

Lung volume (Forced Vital Capacity) and flow rates (Forced Expiratory Volume in 1 second, ratio of Forced Expiratory Volume in 1 second to Forced Vital Capacity (FEV1/FVC) and Peak Expiratory Flow (PEF) were measured using a Spirometer (Micro1; Carefusion). The assessment protocol followed the ATS/ERS task force standardisation of lung function testing guidelines [30]. Normative values were sourced from the European Respiratory Society (ERS) task force Global Lung Function Initiative (GLI) predicted values [31] for FVC, FEV1 and FEV1/FVC. The National Health and Nutrition Examination Survey (NHANES)normal values [32] were used for PEF. The rationale for using these specific normative values was to allow accurate ethic comparison. Normative values provide a generalisable standard for reporting and interpretation of lung volume measurements. Interpretation of pulmonary function tests (PFTs) involves the classification of observed values as within/outside the normal range based on a reference population of healthy individuals.

#### Respiratory muscle strength assessment

Inspiratory Muscle Strength (MIP), Expiratory Muscle Strength (MEP) and Sniff nasal inspiratory pressure (SNIP) were measured using a pressure transducer (MicroRPM; Carefusion) and standardised using the ATS/ERS Statement on Respiratory Muscle Testing. For comparison with normative data, allowing for accurate ethic comparison, male and female normative values for MIP, MEP [33] and SNIP [34], were obtained.

#### Cough assessment

Peak Cough Flow (PCF), a measure of gross respiratory function (strength, flow rate, and respiratory hygiene composite measure) was measured using a PCF meter (Vitalograph & facemask). As there is no standardised testing guideline for PCF, the test was standardised by having participants in standing with the facemask placed over their nose and mouth by the investigator and instructed to “take a breath in and then cough as strongly as you can” following a demonstration by the examiner. The best of 3 attempts was documented as the PCF. For comparison with normative data, a PCF of 360+l/min was considered to be an effective cough, in line with the published literature [35, 36]

#### Cardiovascular fitness assessment

VO2 max was estimated using the YMCA submaximal cycle ergometry test protocol [37], following the ACSM protocol [38]. Participants sat on an upright cycling ergometer (Lode medical ergometer, corival cpet) and pedalled for 3 minutes at zero resistance as a warm-up. Initial work rate was then set at 50 watts for 3 minutes and increased 3 minutes later (minute 6 of the testing protocol) to 70W. Participants pedalled at this resistance for a further 3 minutes, followed by a 3-minute cool down pedalling period. HR, BP and SaO2 were recorded at each 3-minute time point during testing. VO2 max was estimated from the regression line generated between work rate and heart rate responses at each stage. The maximum HR (220-age) was used to estimate what the maximal work rate would be. This work rate was converted to VO2 max (estimated) using the ACSM equation (VO2 (ml.kg^-1^.min^-1^) = 7 + (1.8 x work rate) / body mass (wt. in kg)) for cycle ergometry. Normative reference range for non-athletic males and females by age group were used as the comparator value [39], with the higher value applied. The Six Minute Walk Test (6MWT) was employed to assess functional cardiovascular fitness. This was standardised following the American Thoracic Society (ATS) protocol for the six-minute walk test [40], where participants were asked to walk as far as they could along a marked 30m quiet corridor. The following predictive equations derived age and gender matched normative values: males: 6MWT (m) = 867 - (5.71 x age in years) +(1.03 x height in cm), females: 6MWT (m) = 525 - (2.86 x age in years) + (0.71 x height in cm)–(6.22 x BMI) [41].

### Integrative exercise programme

The duration of the exercise programme tested was 12-weeks with three classes per week: a functional circuit-based training class, a High-intensity Interval Training (HIIT) session using a spin class facility and, a Tai Chi class as detailed in Table 1. Each class ran for one hour and consisted of a warm-up, specific exercise intervention and cool down. All classes were taught and/or supervised by a certified exercise instructor and clinician (i.e., physiotherapist or occupational therapist), with modifications and extra assistance available for anyone who needed them. Attendance at each session was recorded by the instructor.

**Table 1:** Description of exercise classes.

FITT Principle	Description	Progression
F: Once per week I: Moderate T: 60mins T: Functional Training, Circuit class with 10 exercise stations	A class consisting of a warm-up, cool down and exercises to improve performance of activities of daily living. Exercises focused on lower limb, upper limb, and abdominal strength training–using body weight, dumbbells or weighted ankle and wrist straps, standing balance exercises using foam blocks and agility exercises including agility ladders on the floor, throwing balls, walking while carrying a glass of water, zigzagging between cones. The rest time between exercises was 2 mins.	Exercises were progressed on an individual basis over the 12-week period, through either increased weight, repetitions, speed of execution or level of difficulty.
		The exercises also changed across three phases a) an initial adaptation phase with intensive instruction; b) an improvement phase where everyone was supported to improve either technically or in terms of loading; c) a challenge phase (final 4 weeks) where the circuit was changed to become more challenging for all participants.
F: Once per week I: High T: 60mins T: High Intensity Interval Training, Spinning	A workout using stationary exercise bikes, with high intensity efforts sitting and standing on the bike interspersed with low intensity recovery periods.	The intensity and duration of these sessions were progressed over time. Participants started with 25 seconds exercise and 25 seconds rest, building up to 60 seconds on and 60 seconds rest by week 12. Participants were invited to record their maximal revolution per minute for one minute at the end of each class on the lightest gear setting, and this reading was used to guide their effort in the following week’s class, with some flexibility incorporated.
F: Once per week I: Low T: 60mins T: Neurodynamic, Tai Chi	A Chinese martial art consisting of sequences of slow controlled movements, focusing on flexibility, balance and lower limb strengthening. Breathing cues i.e., deep breaths in and out coordinated with movement are also an important feature of this modality.	The class was progressed with respect to the complexity of the routines performed by the participants, including more challenging balancing movements.

### Statistical analysis

Codified data, collected on a proforma were inputted to SPSS (v27) for analysis. Descriptive statistics (mean (SD), median (range) and n (%)) summarised baseline data. Independent t-tests explored differences in baseline respiratory measures against normative data for parametric data and Mann Whitney U tests for non-parametric data. A mixed ANOVA examined whether change in respiratory measures over time was affected by H&Y stage. Where no effect for H&Y stage was observed, the ANOVA was rerun reporting change overtime without H&Y. A repeated measures ANOVA was performed to examine the difference in measures over the three time points, pre-intervention, post intervention and at follow up. Post hoc analysis, with Bonferroni correction, examined changes between the different time points. Where results indicate a large variation in class attendances, further analysis examining the effects of attendance rates will be conducted.

## Results

### Participant demographics

Twenty-three volunteers by self-selection participated in this study. No participant dropped out of the exercise programme; two participants were excluded from the spinning classes due to abnormal haemodynamic responses to aerobic exercise identified during pre-screening. Table 2 summarises the baseline characteristics of all participants and identifies their stages of PD using the Hoehn and Yahr scale.

**Table 2:** Baseline characteristics of participants.

**Patient (N)**	23		
**Age in years (mean+/-SD)**	65.52+/-7.489		
**Gender (Male/Female)**	14/9		
**BMI (Mean+/-SD)**	24.68+/-3.725		
**Disease Duration (Range, mean+/-SD)**	1–19 Years, 6.39+/-5.375		
**On Levodopa Treatment (N)**	23		
**Smokers/Non-smokers/Ex-smokers (N)**	0/10/13		
**History of Respiratory Condition (N)**	1		
**History of Pneumonia/Chest Infection (N)**	4		
**Medication phase during assessment**	ON Phase– 23		
	OFF Phase—0		
**Hoehn & Yahr Scale**	**Total**	**Males**	**Females**
• Stage 0 (N)	1	1	0
• Stage 1 (N)	10	7	3
• Stage 2 (N)	10	4	6
• Stage 3 (N)	2	2	0
• Stage 4 (N)	0	0	0
Median H&Y	2	1	2
**MDS-UPDRS Score**	Mean+/-SD		
Part I: Non-Motor Aspects of Experiences of Daily Living (out of 16)	7.8+/-5.4		
Part II: Motor Aspects of Experiences of Daily Living (out of 52)	8.4+/-6.7		
Part III: Motor Examination (out of 108)	24.5+/-12.7		
Part IV: Motor Complications (out of 23)	2.7+/-3.4		
Total	43.5+/-22.4		

N = number of participants, SD = standard deviation, BMI = Body Mass Index

MDS-UPDRS–Movement Disorders Society Unified Parkinson’s Disease Rating Scale.

### Comparison of baseline respiratory measures and published normative data

When baseline respiratory measures were compared to normative data, Table 3, the PD study participants, had significantly lower values in respiratory muscle strength both inspiratory (MIP and SNIP) and expiratory (MEP). Cough efficiency as measured by PCF was significantly lower in those with PD. Mean PCF for the PD group (279l/min) was well below the 360+l/min threshold for an effective cough and approaching 270l/min, a documented threshold value to prevent pneumonia in other neuromuscular disorders [35, 42]. Spirometry measures of FEV(1); FVC(1) and PEF were also significantly lower in PD than the published normative data. Similarly lower and significant differences were observed in PD for the 6MWT, a measure of functional ability and exercise tolerance.

**Table 3:** Comparison of baseline respiratory measures in PD with normative data.

	PD	Normative	P value	Effect Size	Male PD	Male Normative	P value	Effect Size	Female PD	Female Normative	P value	Effect Size
**Spirometry**												
**FEV1 (l)**	2.5+/-0.7	2.9+/-0.7	0.04*	0.6	2.6+/-0.8	3.3+/-0.6	0.02*	1.7	2.36+/-0.24	2.37+/-0.5	0.94	-0.034
**FVC (l)**	3.1+/-0.9	3.7+/-0.8	0.02*	0.7	3.4+/-0.7	4.2+/-0.7	0.01*	1	2.83+/-0.6	3.05+/-0.265	0.33	0.47
**FEV1/FVC (%)**	82+/-15.8	77.5+/-1.2	0.182	-0.4	80.8+/-19.3	76.9+/-1.2	0.45	-0.28	78.4+/-0.5	84+/-8.8	0.09	-0.882
**PEF (l/min)**	329+/-100	745+/-162	<0.001*	3.06	356+/-121	841.3+/-135	<0.001*	3.7	291.9+/-42	596.3+/-37.5	<0.001*	7.6
**Respiratory Muscle Strength**												
**MIP (cmH**_**2**_ **0)**	45.1+/-24.2	89.5+/-15.7	<0.001*	2.1	49+/-25.4	98.5+/-11.8	<0.001*	2.5	39.5+/-22.6	75.5+/-9.5	<0.001*	2
**MEP cmH**_**2**_ **0)**	72+/-27.7	112.4+/-26.4	<0.001*	1.49	76.8+/-31.2	130.7+/-15.2	<0.001*	2.2	64.7+/-20.7	83.9+/-5.8	0.025*	1.3
**SNIP (cmH**_**2**_ **0)**	40.7+/-24.5	89.6+/-11.3	<0.001*	2.5	46.4+/-26.2	92.5+/-13	<0.001*	2.2	32.2+/-19.8	85+/-6.4	<0.001*	3.6
**Peak Cough Flow**												
**PCF (l/min)**	279+/-113	360+/-0	0.002*	1.09	286.8+/-132.7	360+/-0	0.05*	0.78	268.1+/-78.6	360+/-0	0.003*	1.6
**Cardiovascular Fitness**												
**Estimated VO**_**2**_ **max (l/min)**	32.9+/-12.9	34.6+/-1.5	0.57	0.18	29.2+/-4.2	38.14+/-4.9	<0.001*	1.9	38.8+/-19.4	30+/-0	0.24	-0.67
**6MWT (m)**	437+/-110	656.5+/-55.6	<0.001*	2.5	449+/-125	674.3+/-60	<0.001*	2.3	419.6+/-86	628.7+/-35	<0.001*	3.2

Data are expressed as mean and SD. FEV1: Forced Expiratory Volume in 1 second, FVC: Forced Vital Capacity, PEF: Peak Expiratory Flow, MIP: Maximum Inspiratory Pressure, MEP: Maximum Expiratory Pressure, SNIP: Sniff Nasal Inspiratory Pressure, PCF: Peak Cough Flow, l:litre, l/min: litre per minute, cmH2O: centimetres of water

*:p ≤0.05

### Effects of an integrated exercise programme on respiratory outcomes

The attendance rate at scheduled classes was high, despite the element of choice. The 2 participants who were excluded from the spin classes due to abnormal haemodynamic response to exercise identified during pre-screening were not considered in attendance numbers. As high attendance at all classes was noted 84% overall, (Tai Chi 84%, Functional Training 87%, Spinning 81%), planned sub-analysis on the impact of class attendance on outcomes was not conducted.

The mixed ANOVA identified no significant effect for H&Y stage on the performance of measures over time; therefore, results are reported over time without H&Y stage consideration. Table 4 summarises the pre, post and follow-up results for change over time in all outcome measures of interest using a repeated measure ANOVA.

**Table 4:** Outcome measures pre-post intervention.

	Pre-intervention	Post Intervention	16 weeks Post Intervention	F	Df	P	Partial ETA squared
**MDS-UPDRS Scores**							
**Medication State**	On—23	On -21	On -21	-	-	-	-
		Off-2	Off-1				
**Total MDS-UPDRS Scores**	45.4+/-20.9	40.3+/-22.8	35.7+/-25.5^++^	5.4	2	0.008*	0.2
**MDS-UPDRS Part III**	24.5+/-12.7	22.2+/-13.6	15.9+/-15.2	8.5	2	<0.001*	0.3
**Respiratory Rate**							
**RR (bpm)**	17.1(2.7)	18(3.5)	17.4(2.4)	1.3	2	0.28	0.06
**Spirometry**							
**FEV1 (l)**	2.4 (0.7)	2.4 (0.7)	2.6 (0.6)	1.9	2	0.16	0.08
**FVC (l)**	3.2 (0.9)	2.8 (0.8)	2.9 (0.5)	0.8	2	0.45	0.04
**FEV1/FVC (%)**	82.1 (15.6)	85.7 (10.6)	88.9 (8.6)	2.7	2	0.08	0.11
**PEF (l/min)**	320.9 (93.5)	368.0 (112.2)	371.1 (113.7)	3.6	2	0.04*	0.15
**Respiratory Muscle Strength**							
**MIP (cmH**_**2**_ **0)**	43.9 (25.0)	48.9(23.7)	43.4 (23.6)	1.4	2	0.26	0.62
**MEP (cmH**_**2**_ **0)**	71.3 (28.1)	85.1 (26.8)^**+**^	71.5 (29.1) **--**	5.6	2	0.007*	0.20
**SNIP (cmH**_**2**_ **0)**	42.0 (24.4)	47.8 (24.1)	37.2 (15)	2.6	2	0.09	0.11
**Cough Flow**							
**PCF (l/min)**	278.5 (115.5)	270.4 (85.1)	259.1 (89.3)	0.4	2	0.669	0.02
**Cardiovascular Fitness**							
**Estimated VO**_**2**_ **max (l/min)**	30+/-15.5	31.2+/-8.5	30.6+/-7.5	1.3	1.6	0.28	0.06
**6MWT (m)**	426.5+/-99.0	501+/-85.8^**+**^	479.4+/-93.3^**--**^	19.7	1.5	<0.001*	0.50

Data are expressed as mean and SD. FEV1: Forced Expiratory Volume in 1 second, FVC: Forced Vital Capacity, PEF: Peak Expiratory Flow, MIP: Maximum Inspiratory Pressure, MEP: Maximum Expiratory Pressure, SNIP: Sniff Nasal Inspiratory Pressure, PCF: Peak Cough Flow

*P ≤0.05. Bonferroni adjusted post hoc testing: +significant improvement post intervention p<0.01, ++ significant improvement from baseline to long term follow up p<0.005,—deteriorated from post intervention to follow-up p = 0.05.

A statistically significant difference, with a large effect size, was noted in Total MDS-UPDRS and MDS-UPDRS Part III scores over time, with significant and incremental improvement evident at each timepoint. Statistically significant and incremental improvements in PEF were also observed over time with large effect size (MD: 50.2, 95% CI: 2.8, 97.5, p = 0.04). While MEP and 6MWT outcomes were observed to change over time with large effect size, post hoc analysis using Bonferroni adjustment identified that MEP (MD: 13.8, 95% CI: 2.5, 25.1, p = 0.013) and 6MWT (MD: 74.4, 95% CI: 40.6, 108.2 p<0.001) significantly increased from baseline to post intervention but significantly decreased between this and 16 week follow up (MD: -13.5, 95% CI: -27, -0.07, p = 0.048 and (MD: -21.5, 95% CI: -42.7, -0.32, p = 0.046 respectively).

Post intervention improvements were noted in the outcomes of FEV1/FVC, MIP, SNIP and VO2max. These did not reach statistical significance and showed large (MIP) and medium (FEV1/FVC, SNIP, VO2max) effect sizes. The measures of FVC, FEV1 and PCF did not show improvement post intervention, rather values decreased over time.

## Discussion

This study, which tested an opt-in, community based integrative exercise programme comprising three different types of exercise classes in PD, demonstrated statistically significant reductions in MDS-UPDRS Scores over the 12 weeks, that were sustained at follow-up. This was not an unexpected finding given evidence from both human and animal models that exercise has a neuroprotective role and can improve motor impairments and physical condition in people with PD regardless of disease staging [43–45]. Measuring respiratory function in PD is not standard practice despite evidence of obstructive, restrictive, mixed, and central issues reported in the literature [13]. Similarly, inclusion of outcomes addressing the spectrum of respiratory dysfunction is not routine in exercise trials in PD, with limited evidence to support their efficacy to affect improvements in respiratory function [26]. This study identified significant baseline impairments in respiratory muscle strength (inspiratory and expiratory), PCF rates and cardiovascular fitness (6MWT) and abnormalities in spirometry measures addressing restrictive (FVC) and obstructive (PEF) dysfunction, in participants presenting to a community-based exercise class for PD. These are interesting findings in the context of the study population where the majority are in the early stages of disease progression (87% in H&Y stages 1–2), although preclinical studies have previously highlighted early respiratory impairments in PD [46, 47]. The 12-week opt-in integrative exercise programme targeting resistance training and balance (functional circuit training classes and tai chi) and aerobic training (spin classes) affected short term, statistically significant improvements in expiratory muscle strength, PEF and the 6MWT. Non-significant improvements were noted in inspiratory muscle strength and estimated Vo2max. Improvements were not maintained long term (except for PEF rate), suggesting ongoing input is required for maintenance. Resting respiratory rate remained largely unchanged and PCF rate was noted to decline over the testing period. Elements of respiratory dysfunction identified in this study appear to be responsive to exercise in people with PD in the short term. While the intervention tested in this study allowed participants freedom of choice in their exercise program, all participants attended a high proportion (>82%) of all classes. It was not possible to examine further which exercise component/s may have offered greatest benefit to the respiratory metrics which improved post-intervention.

Significant respiratory muscle weakness was evident in the PD group compared to normative values in this study for both inspiratory and expiratory muscle groups. Expiratory muscle weakness is well established in published PD literature [10, 48–50] and non-pharmacological interventions have been shown by meta-analysis to improve MEP by 19cmH_2_0 (CI 7.79–30.14) [26] with the main effect observed when expiratory muscle strength training is targeted [24, 51]. Evidence from other populations supports moderate to vigorous intensity aerobic exercise affects improvements in expiratory muscle strength [52–54]. This study now demonstrates for the first time in PD, an integrated exercise programme, that included a high intensity aerobic spin class, affected change in expiratory muscle groups. Increased MEP may be because of improved thoracic expansion from Tai Chi, or the documented global effort exercise, HIIT, and circuit based functional training has on strengthening the expiratory muscles. Inspiratory muscle weakness, also well documented in PD [50, 55–57] and other degenerative neurological conditions [58, 59] is responsive to exercise interventions, noted in a meta-analysis of strength training [60], exergaming [61] and functional exercises [61] to improve MIP by 25.85cmH_2_0 (CI 8.34–43.36). This current study echoes these results with large effect sizes observed for both MIP and SNIP improvement following intervention. While these findings did not achieve statistical significance, results may be a result of inadequate sample size power or point to greater benefit accrual from more specific inspiratory muscle training programmes [62] or resistance-based strength training interventions, also shown to increase inspiratory muscle activation with increased effort and abdominal strengthening [60].

Motor impairments of cough, asymptomatic in up to 50% of people with PD, have been reported in early disease stages, with both motor and sensory deficits prevalent in the later stages [63–65]. In this study, baseline values recorded for voluntary cough flow were low and deteriorated over the course of the study, indicating the predominantly early-stage PD participants are at risk of infection due to ineffective airways clearance. PCF values in excess of 360l/min are considered normal in a healthy population [66], with excess of 180l/min required for effective mucus removal and prevention of pneumonia in neuromuscular diseases [67]. The notable dystussia identified in participants is important to note in PD where respiratory disorders are the most common cause of death [2] and account for a third of all hospital admissions [5]. Furthermore, reflex, and voluntary cough dysfunction is often concomitant with dysphagia in people with PD [68–70]. Addressing cough impairment is not straightforward, as it can include motor and sensory aspects of impairment. Differences exist in the literature as to the most effective respiratory muscles to improve PCF, with some reporting a higher correlation between inspiratory muscle strength and PCF for example in multiple sclerosis [71], whereas others report improved cough function following expiratory muscle training in populations with PD [24], stroke and other conditions [72–74]. Improvements in respiratory muscle strength (MIP and MEP) have previously been associated with improved motor components measured as PCF [75, 76], yet, despite improvements in MEP and MIP evident in this study post intervention, PCF values continued to deteriorate over the course of the study.

Spirometry values, including PEF rate, were significantly lower in the PD group compared with published normative data, however FEV1/FVC ratios suggested no airways obstruction. Conflicting evidence again exists in relation to the pattern of respiratory dysfunction, with the prevalence of restrictive dysfunction varying from 28–94% and obstructive varying from 6.7–67% in the literature [13]. Recent meta-analyses point to a restrictive pattern, indicated by reduced FVC and FEV1, and potentially caused by evident inspiratory muscle weakness [14] like findings in this study. The lower PEF rates observed are not in keeping with pooled meta-analytics previously published [14] and have no obvious explanation as a measure reflecting large airways patency. Significant improvement in PEF rates were seen post intervention, across all timepoints in this current study in contrast to all other measures. While it is not anticipated that the exercise intervention would have affected airway patency, PEF starts after a maximum inspiration and records how hard and fast one can forcefully exhale. Therefore, improvements in thoracic expansion from Tai Chi, or inspiratory and expiratory muscle strength may have influenced PEF where HIIT, and circuit based functional training may have contributed. PEF has been previously shown to improve significantly in people with PD following general exercise-based interventions [26], a finding also seen in healthy individuals and those with asthma [77–79]. The effort required to produce PEF is heavily influenced by muscle strength [80, 81] and this measure may improve further with interventions which specifically target respiratory muscle strengthening. The lack of improvement in other spirometry findings are more clearly echoed in other studies investigating the effects of a non-specific exercise intervention on respiratory metrics in people with PD [61, 82, 83]. Exercise interventions which include a specific respiratory strength training component do appear to have positive outcomes on some other aspects of spirometry function, namely FVC [60, 82, 84].

While measures of central pathogenesis of respiratory dysfunction in PD including sensitivity to oxygen and carbon dioxide levels were not feasible to perform in this study, mean resting respiratory rate recorded (17breaths/min) was higher than the normal range (12–16 breaths/min) [85] although not outside the range for older adults [86]. Pre-clinical studies on rats have demonstrated a central respiratory disorder where changes in respiratory rate and altered response to hypoxia and hypercapnia emerge soon after a lesion evoking degeneration of dopaminergic neurons [87, 88]. Mechanisms for the increased respiratory rate in PD include the presence of neurodegeneration in brainstem central respiratory centres causing central pacing and chemosensitivity issues and diaphragmatic dyskinesis due to levodopa medication [13]. The increased RR was not responsive to the exercise intervention despite evidence in healthy and sedentary populations of a reduction in resting respiratory rate after prolonged exercise when compared with matched controls [89, 90].

Estimated VO2max was reduced in the PD group compared to published norms. This metric, while improved post intervention, did not reach statistical significance, similar to previously published findings in PD [91]. This may result from an insufficient aerobic training dosage to bring about a cardiovascular training response in the integrated exercise programme [92] or from low study numbers with insufficient power. Disaggregation of VO2 max by sex, Table 3 identified differences, whereby estimated VO2 max was higher than normative data in females, most likely driven by one outlier in the group. In contrast and similar to other published research [93], estimated VO2max was significantly reduced in male participants at baseline. Submaximal tests, as used in this study, are known to overestimate true VO2max [38] which suggests cardiovascular fitness in PD may be more reduced than findings presented. Mean estimated VO2max reported in Table 2 is notably higher than findings published in a systematic review of aerobic capacity in PD (22.2 mL O_2_·kg^−1^·min^−1^ and 21.9 mL O_2_·kg^−1^·min^−1^ in ON and OFF phases respectively [94]). In addition, contrary to findings presented in this study, the systematic review reported no differences between individuals with PD and healthy controls in their cardiovascular fitness. One explanation for this discrepancy in findings may be many resources cite reference ranges rather than absolute values as normative data for V02max, therefore direct comparison with matched controls may be more reliable. Baseline 6MWT results compared to normative data, confirm significant loss of functional fitness capacity in PD which appears to be responsive to the integrated exercise programme. Improvement in 6MWT is most likely related to HIIT as demonstrated in other conditions [95, 96] however strength training [97] and Tai Chi [98] may also have contributed. Different modes of exercise have previously been proven to improve 6MWT distance including walking [99], aerobic exercise [100], dance [101] and resistance training [102]. Cardiovascular autonomic disturbance in PD is another factor to be considered in aerobic training in PD. This study identified an altered cardiovascular response to aerobic exercise in two participants during screening. This finding suggests careful screening and monitoring of patients is essential [103]. Endurance training has been found to be beneficial in alleviating cardiovascular autonomic dysfunction in PD, but further research is required [102].

Significant short-term improvements were observed in MEP, 6MWT and PEF. In the absence of continuing exercise, benefits were not maintained for MEP or 6MWT. Exercise in progressive neurological conditions such as PD, has been shown to benefit both motor and nonmotor signs, improve physical function and reduce disability [104]. However, ongoing maintenance programmes are required, to maintain improvements [105] and reduce the rate of decline [106, 107] Therefore sustainable exercise programmes aiming to provide long term improvement in and/or maintenance of respiratory measures in PD are needed.

The authors acknowledge a number of limitations in this research. Due to the small sample size, the study may not be adequately powered to detect statistically significant change in measures which may have improved following the programme of exercise. The smaller sample size also has implications on the generalizability of study findings. Sampling bias is possible based on the non-probability sampling method employed and the opt-in nature of the selection process may lead to self-selection bias, where participants may be more receptive to exercise and have prior experience exercising. In the absence of a control group, changes in respiratory metrics cannot be directly attributed to the exercise intervention with certainty. Most participants in this study had mild PD symptoms (H&Y 0–2) therefore the findings presented at baseline and in response to exercise may not be representative of all PD patients. Effect sizes for all changes in measures observed were medium or large, showing promise as preliminary findings. Further research is now warranted in larger, adequately powered, and controlled studies across all stages of PD. Although perceived dyspnoea was not included in the current study as an outcome of interest, given the presence of increased perception of dyspnoea in the PD population [13], it’s impact on quality of life and its multifactorial nature, with central peripheral and emotional processes hypothesised to be involved [88], inclusion of it as a measure in future trials is strongly recommended. All participants were taking levodopa medication and were in the ON phase during testing. Levodopa medication has been found to have both positive; improved FVC, FEV1, Vital Capacity (VC), PEF, and respiratory muscle strength, and negative effects; dyspnoea, tachypnoea, hyperventilation and dyskinesias. on the respiratory system. There are conflicting data on the effects of exercise on levodopa, where both increases [108–110] and decreases [108, 110] in levodopa absorption following exercise have been demonstrated. Given this, the increase in PEF, MEP and 6MWT following exercise may be an indirect effect of improved levodopa absorption leading to changes rather than direct effects of exercise. Further research in both the ON and OFF phases and in patients who are not taking levodopa medication is also recommended.

This study explored how an integrative, non-specific exercise programme could identify impairments and enhance respiratory function in PD. This differs from previous studies that employed targeted, clinical intervention/s that could be considered less ecologically valid in comparison to community-based exercise programmes, available to large groups of people outside of a medical setting. To ensure greater numbers of people with PD have access to effective exercise supports, future work is required to determine which sustainable community-based exercise regimes offer the greatest improvement in respiratory function and greatest all-round long-term benefits i.e., cardiovascular fitness, respiratory function, mobility; strength/balance/coordination, enjoyment, social interaction and support, cognitive function.

## Conclusion

This study identifies merit in including baseline and follow-up respiratory outcomes for exercise interventions targeting individuals with PD. Significant respiratory dysfunction exists, even in the early stages of PD. Metrics of respiratory muscle strength, peak expiratory flow rate and functional aerobic capacity appear to be responsive to an opt-in integrative exercise programme. The benefits are not well-preserved following programme completion suggesting additional and ongoing maintenance strategies are required. These findings now warrant further testing in an adequately powered randomised control trial.

## Supporting information

S1 Checklist Strobe checklist. (DOCX)

S1 Data Dataset. (XLSX)

## References

[1] Hovestadt A, Bogaard JM, Meerwaldt JD, van Der Meche FG, Stigt J. Pulmonary function in Parkinson’s disease Journal of Neurology, Neurosurgery & Psychiatry 1989;52(3):329–33. 10.1136/jnnp.52.3.329 PMC1032405 2926415

[2] Pennington S, Snell K, Lee M, Walker R. The cause of death in idiopathic Parkinson’s disease. Parkinsonism Relat Disord. 2010;16(7):434–7. Epub 2010/06/24. doi: 10.1016/j.parkreldis.2010.04.010 . 20570207

[3] Schrag A, M J, N Q. What contributes to quality of life in patients with Parkinson’s disease? Journal of Neurology, Neurosurgery & Psychiatry. 2000;69:308–12. doi: 10.1136/jnnp.69.3.308 10945804 PMC1737100

[4] Müller J, Wenning GK, Verny M, McKee A, Chaudhuri KR, Jellinger K, et al. Progression of dysarthria and dysphagia in postmortem-confirmed parkinsonian disorders. Archives of neurology. 2001;58(2):259–64. doi: 10.1001/archneur.58.2.259 11176964

[5] Kelly B, Blake C, Lennon O. Acute Hospital Admissions of Individuals with a Known Parkinson’s Disease Diagnosis in Ireland 2009–2012. J Parkinsons Dis. 2016;6(4):709–16. doi: 10.3233/JPD-160839 27662326

[6] Low DA, Vichayanrat E, Iodice V, Mathias CJ. Exercise hemodynamics in Parkinson’s disease and autonomic dysfunction. Parkinsonism Relat Disord. 2014;20(5):549–53. Epub 2014/03/19. doi: 10.1016/j.parkreldis.2014.02.006 . 24637120

[7] Gil-Prieto R P-GR, San-Roman-Montero J, Martinez-Martin P, Castrodeza-Sanz J, Gil-de-Miguel A. Measuring the Burden of Hospitalization in Patients with Parkinson´s Disease in Spain. PLoS ONE 2016;11(3): e0151563. 26977930 10.1371/journal.pone.0151563 PMC4792469

[8] Shill H, Stacy M. Respiratory Complications of Parkinson’s Disease. Seminars in Respiratory and Critical Care Medicine. 2002;23(3):261–4. doi: 10.1055/s-2002-33034 16088618

[9] Sabaté M GI, Ruperez F, and Rodríguez M. Obstructive and restrictive pulmonary dysfunctions in Parkinson’s. Journal of the neurological sciences. 1996;138(1–2):114–9. 10.1016/0022-510x(96)00003-2. 8791248

[10] Sanches VS, Santos FM, Fernandes JM, Santos MLM, Müller PT, Christofoletti G. Neurodegenerative disorders increase decline in respiratory muscle strength in older adults. Respiratory Care. 2014;59(12):1838–45. doi: 10.4187/respcare.03063 25205817

[11] Mehanna R, Jankovic J. Respiratory problems in neurologic movement disorders. Parkinsonism Relat Disord. 2010;16(10):628–38. Epub 2010/08/03. doi: 10.1016/j.parkreldis.2010.07.004 . 20674459

[12] O’Callaghan A, Walker R. A review of pulmonary function in Parkinson’s disease. Journal of Parkinsonism and Restless Legs Syndrome. 2018;Volume 8:13–23. doi: 10.2147/jprls.S114309

[13] D’Arrigo A, Floro S, Bartesaghi F, Casellato C, Papa GF, Centanni S, et al. Respiratory dysfunction in Parkinson’s disease: a narrative review. ERJ Open Research. 2020;6(4). doi: 10.1183/23120541.00165-2020 33043046 PMC7533305

[14] McMahon L, Blake C, Lennon O. A systematic review and meta-analysis of respiratory dysfunction in Parkinson’s disease. Eur J Neurol. 2023;30(5):1481–504. Epub 2023/02/14. doi: 10.1111/ene.15743 . 36779856

[15] Toole T, Hirsch M.A, Forkink A, Lehman D.A, Maitland C.G. The effects of a balance and strength training program on equilibrium in Parkinsonism: a preliminary study. NeuroRehabilitation. 2000;14:165–74. 11455079

[16] Hirsch M.A, Toole T, Maitland C.G, Rider R.A. The effects of balance training and high-intensity resistance training on persons with idiopathic Parkinson’s disease. Arch Phys Med Rehabil. 2003;84:1109–17. doi: 10.1016/s0003-9993(03)00046-7 12917847

[17] Uhrbrand A.S.E, Pedersen M.S, Dalgas U. J. Parkinson’s disease and intensive exercise therapy–a systematic review and meta-analysis of randomized controlled trials. Neurol Sci 2015;15:9–19. doi: 10.1016/j.jns.2015.04.004 25936252

[18] Corcos DM, Robichaud JA, David FJ, Leurgans SE, Vaillancourt DE, Poon C, et al. A two-year randomized controlled trial of progressive resistance exercise for Parkinson’s disease. Mov Disord. 2013;28(9):1230–40. Epub 2013/03/29. doi: 10.1002/mds.25380 ; PubMed Central PMCID: PMC3701730. 23536417 PMC3701730

[19] Ellis T, de Goede C.J, Feldman R.G, Wolters E.C, Kwakkel G, Wagenaar R.C. Efficacy of a physical therapy program in patients with Parkinson’s disease: a randomised controlled trial. Arch Phys Med Rehabil 2005;86:626–32. 15827910 10.1016/j.apmr.2004.08.008

[20] Schenkman M, Cutson TM, Kuchibhatla M, Chandler J, Pieper C.F, Ray L, et al. Exercise to improve spinal flexibility and function for people with Parkinson’s disease: a randomized, controlled trial. J Am Geriatr Soc (JAGS). 1998;46:1207–16. doi: 10.1111/j.1532-5415.1998.tb04535.x 9777901

[21] Roeder L.C.J, Smith S.S, Stewart I.B, Kerr G.K. Effects of resistance training on measures of muscular strength in people with Parkinson’s disease: a systematic review and meta-analysis. PLoS One. 2015;10. doi: 10.1371/journal.pone.0132135 26146840 PMC4492705

[22] Ribeiro R, Brandao D, Noronha J, Lima C, Fregonezi G, Resqueti V, et al. Breath-stacking and incentive spirometry in Parkinson’s disease: Randomized crossover clinical trial. Respir Physiol Neurobiol. 2018;255:11–6. Epub 2018/05/05. doi: 10.1016/j.resp.2018.04.011 . 29727719

[23] Kuo YC, Chan J, Wu YP, Bernard JR, Liao YH. Effect of expiratory muscle strength training intervention on the maximum expiratory pressure and quality of life of patients with Parkinson disease. NeuroRehabilitation. 2017;41(1):219–26. Epub 2017/05/21. doi: 10.3233/NRE-171474 . 28527233

[24] Reyes A, Castillo A, Castillo J, Cornejo I. The effects of respiratory muscle training on peak cough flow in patients with Parkinson’s disease: a randomized controlled study. Clin Rehabil. 2018;32(10):1317–27. Epub 2018/05/15. doi: 10.1177/0269215518774832 . 29756459

[25] Reyes A, Castillo A, Castillo J, Cornejo I, Cruickshank T. The Effects of Respiratory Muscle Training on Phonatory Measures in Individuals with Parkinson’s Disease. J Voice. 2019;31:S0892–1997. Epub 2019/06/04. doi: 10.1016/j.jvoice.2019.05.001 . 31155431

[26] McMahon L, Blake C, Lennon O. Nonpharmacological interventions for respiratory health in Parkinson’s disease: A systematic review and meta-analysis. European Journal of Neurology. 2020;(n/a). doi: 10.1111/ene.14605 33098349

[27] McGrath D ML, Walsh A, Cunningham C, Lennon O., editor. A feasibility study for a university-based community exercise programme for people with Parkinson’s Disease. 23rd Congress European College of Sports Science; 2018 6 Jul 2018.

[28] Goetz CG, Fahn S, Martinez-Martin P, Poewe W, Sampaio C, Stebbins GT, et al. Movement Disorder Society-sponsored revision of the Unified Parkinson’s Disease Rating Scale (MDS-UPDRS): Process, format, and clinimetric testing plan. Movement Disorders. 2007;22(1):41–7. doi: 10.1002/mds.21198 17115387

[29] Martinez-Martin P, Rodriguez-Blazquez C, Mario A, Arakaki T, Arillo VC, Chana P, et al. Parkinson’s disease severity levels and MDS-Unified Parkinson’s Disease Rating Scale. Parkinsonism Relat Disord. 2015;21(1):50–4. Epub 2014/12/04. doi: 10.1016/j.parkreldis.2014.10.026 . 25466406

[30] Miller MR, Hankinson J, Brusasco V, Burgos F, Casaburi R, Coates A, et al. Standardisation of spirometry. European Respiratory Journal. 2005;26(2):319–38. doi: 10.1183/09031936.05.00034805 16055882

[31] Quanjer PH, Stanojevic S, Cole TJ, Baur X, Hall GL, Culver BH, et al. Multi-ethnic reference values for spirometry for the 3–95-yr age range: the global lung function 2012 equations. European Respiratory Journal. 2012;40(6):1324–43. doi: 10.1183/09031936.00080312 22743675 PMC3786581

[32] Hankinson JL, Odencrantz JR, KB F. Spirometric reference values from a sample of the general U.S. population. Am J Respir Crit Care Med. 1999;159:179–87. doi: 10.1164/ajrccm.159.1.9712108 9872837

[33] Evans JA, Whitelaw WA. The assessment of maximal respiratory mouth pressures in adults. Respir Care. 2009;54(10):1348–59. Epub 2009/10/03. . 19796415

[34] Uldry C, Fitting JW. Maximal values of sniff nasal inspiratory pressure in healthy subjects. Thorax. 1995;50(4):371–5. Epub 1995/04/01. doi: 10.1136/thx.50.4.371 ; PubMed Central PMCID: PMC474280. 7785009 PMC474280

[35] Bach JR, Ishikawa Y, Kim H. Prevention of pulmonary morbidity for patients with Duchenne muscular dystrophy. Chest. 1997;112(4):1024–8. Epub 1997/10/23. doi: 10.1378/chest.112.4.1024 . 9377912

[36] Leith DE. The Development of Cough. American Review of Respiratory Disease. 1985;131(S5):S39–S42. doi: 10.1164/arrd.1985.131.S5.S39 . 4003907

[37] Golding L, Myers CR, Sinning W. Y’s Way to Physical Fitness: The Complete Guide to Fitness Testing and Instruction. 3rd ed. USA: YMCA of the USA; 1989. 191 p.

[38] Greiwe JS, Kaminsky L. A., Whaley M. H. & Dwyer G. B. Evaluation of the ACSM submaximal ergometer test for estimating V̇O2max. Medicine & Science in Sports & Exercise. 1995;27 (9):1315–20. 8531631

[39] Kenney WL, Wilmore JH, Costill DL. Physiology of sport and exercise. Seventh ed. Champaign, IL: Human Kinetics; 2020.

[40] ATS statement: guidelines for the six-minute walk test. Am J Respir Crit Care Med. 2002;166(1):111–7. Epub 2002/07/02. doi: 10.1164/ajrccm.166.1.at1102 . 12091180

[41] Jenkins S, Cecins N, Camarri B, Williams C, Thompson P, Eastwood P. Regression equations to predict 6-minute walk distance in middle-aged and elderly adults. Physiother Theory Pract. 2009;25(7):516–22. Epub 2009/11/21. doi: 10.3109/09593980802664711 . 19925174

[42] Finder JD, Birnkrant D, Carl J, Farber HJ, Gozal D, Iannaccone ST, et al. Respiratory care of the patient with Duchenne muscular dystrophy: ATS consensus statement. Am J Respir Crit Care Med. 2004;170(4):456–65. Epub 2004/08/11. doi: 10.1164/rccm.200307-885ST . 15302625

[43] Hirsch MA, Farley BG. Exercise and neuroplasticity in persons living with Parkinson’s disease. Eur J Phys Rehabil Med. 2009;45(2):215–29. Epub 2009/06/18. . 19532109

[44] Kim JD, McCarter RJM, Yu BP. Influence of age, exercise, and dietary restriction on oxidative stress in rats. Aging Clinical and Experimental Research. 1996;8(2):123–9. doi: 10.1007/BF03339566 8737611

[45] Reuter I, Engelhardt M, Stecker K, Baas H. Therapeutic value of exercise training in Parkinson’s disease. Med Sci Sports Exerc. 1999;31(11):1544–9. Epub 1999/12/10. doi: 10.1097/00005768-199911000-00008 . 10589855

[46] de Campos PS, Kawamura LRSM, Hasegawa K, Kumei Y, Zeredo JL. Analysis of respiratory movements in a mouse model of late Parkinson’s disease submitted to stress. Respiratory Physiology & Neurobiology. 2018;251:50–6. doi: 10.1016/j.resp.2018.02.012 WOS:000428825500007. 29481879

[47] Lima JC, Oliveira LM, Botelho MT, Moreira TS, Takakura AC. The involvement of the pathway connecting the substantia nigra, the periaqueductal gray matter and the retrotrapezoid nucleus in breathing control in a rat model of Parkinson’s disease. Experimental Neurology. 2018;302:46–56. doi: 10.1016/j.expneurol.2018.01.003 29305892

[48] Santos RBD, Fraga AS, Coriolano MDGWS, Tiburtino BF, Lins OG, Esteves ACF, et al. Respiratory muscle strength and lung function in the stages of Parkinson’s disease. Jornal brasileiro de pneumologia: publicacao oficial da Sociedade Brasileira de Pneumologia e Tisilogia. 2019;45(6):e20180148. doi: 10.1590/1806-3713/e20180148 31576908 PMC7447545

[49] Guedes LU, Rodrigues JM, Fernandes AA, Cardoso FE, Parreira VF. Respiratory changes in parkinson’s disease may be unrelated to dopaminergic dysfunction. Arquivos de Neuro-Psiquiatria. 2012;70(11):847–51. doi: 10.1590/s0004-282x2012001100005 23175196

[50] Sathyaprabha TN, Kapavarapu PK, Pall PK, Thennarasu K, Raju TR. Pulmonary Functions in Parkinson’s Disease. The Indian Journal of Chest Diseases & Allied Sciences. 2005;47(4):251–7. 16255396

[51] Sapienza C, Troche M, Pitts T, Davenport P. Respiratory strength training: concept and intervention outcomes. Seminars in Speech and Language 2011 Feb;32(1):21–30. 2011. doi: 10.1055/s-0031-1271972 21491356

[52] Dassios T, Katelari A, Doundounakis S, Dimitriou G. Aerobic exercise, and respiratory muscle strength in patients with cystic fibrosis. Respir Med. 2013;107:684–90. doi: 10.1016/j.rmed.2013.01.016 23485096

[53] Yamashina Y, Aoyama H, Hori H, Morita E, Sakagami N, Hirayama T, et al. Comparison of respiratory muscle strength in individuals performing continuous and noncontinuous walking exercises in water after the 6-week program. JER. 2019;15(4):566–70. doi: 10.12965/jer.1938260.130 31523678 PMC6732542

[54] Taskin H, Atalay O, Kurtca M, Kabul E, Calik B, Yalman A, et al. The effects of aerobic training on respiratory muscle strength and exercise capacity in ankylosing spondylitis. European Respiratory Soc Journals Ltd; 2018.

[55] Haas BM, Trew M, Castle PC. Effects of respiratory muscle weakness on daily living function, quality of life, activity levels, and exercise capacity in mild to moderate Parkinson’s disease. American Journal of Physical Medicine and Rehabilitation. 2004;83(8):601–7. doi: 10.1097/01.phm.0000133436.61009.02 15277961

[56] Maria B, Sophia S, Michalis M, Charalampos L, Andreas P, John ME, et al. Sleep breathing disorders in patients with idiopathic Parkinson’s disease. Respir Med. 2003;97(10):1151–7. Epub 2003/10/17. doi: 10.1016/s0954-6111(03)00188-4 . 14561023

[57] De Bruin P DBV, Lees A, Pride N,. Effects of Treatment on Airway Dynamics and Respiratory Muscle Strength in Parkinson’s Disease. American Review of Respiratory Disease. 1993;148(6):1576–80. doi: 10.1164/ajrccm/148.6_Pt_1.1576 . 8256904

[58] Mangera Z, Panesar G, Makker H. Practical approach to management of respiratory complications in neurological disorders. Int J Gen Med. 2012;5:255–63. Epub 2012/03/21. doi: 10.2147/IJGM.S26333 . 22505823 PMC3325013

[59] Pfeffer G, Povitz M. Respiratory management of patients with neuromuscular disease: current perspectives. Degener Neurol Neuromuscul Dis. 2016;6:111–8. doi: 10.2147/DNND.S87323 . 30050373 PMC6053085

[60] Alves WM, Alves TG, Ferreira RM, Lima TA, Pimentel CP, Sousa EC, et al. Strength training improves the respiratory muscle strength and quality of life of elderly with Parkinson disease. The Journal of sports medicine and physical fitness. 2019;59(10):1756–62. doi: 10.23736/S0022-4707.19.09509-4 31113177

[61] DeAlmeida. Effects of Physical Training with Exergaming and Functional Training in Individuals with Parkinson’s Disease. [PhD Thesis.]. 2018.

[62] Inzelberg R, Peleg N, Nisipeanu P. Inspiratory muscle training and the perception of dyspnea in Parkinson’s disease. Can J Neurol Sci. 2005;32(2):213–7. doi: 10.1017/s0317167100003991 16018157

[63] Fontana G, Pantaleo T, Lavorini F, Benvenuti F, Gangemi S. Defective Motor Control of Coughing in Parkinson’s Disease. American Journal of Respiratory and Critical Care Medicine. 1998;158(2):458–64. doi: 10.1164/ajrccm.158.2.9705094 9700121

[64] Ebihara S, Saito H, Kanda A, Nakajoh M, Takahashi H, H A, et al. Impaired Efficacy of Cough in Patients with Parkinson Disease. Chest. 2003;124(3):1009–15. doi: 10.1378/chest.124.3.1009 12970031

[65] Fuh JL, Lee RC, Wang SJ, Lin CH, Wang PN, Chiang JH, et al. Swallowing difficulty in Parkinson’s disease. Clin Neurol Neurosurg. 1997;99(2):106–12. Epub 1997/05/01. doi: 10.1016/s0303-8467(97)00606-9 . 9213054

[66] Leith DE. Cough. In: Brain JD, Proctor D, Reid L, editors. Lung biology in health and disease: Respiratory defense mechanisms, part 2. New York: Marcel Dekker; 1977. p. 545–92.

[67] Bach JR. Amyotrophic lateral sclerosis: predictors for prolongation of life by noninvasive respiratory aids. Arch Phys Med Rehabil. 1995;76(9):828–32. Epub 1995/09/01. doi: 10.1016/s0003-9993(95)80547-8 . 7668953

[68] Pitts T, Bolser D, Rosenbek J, Troche M, Sapienza C. Voluntary cough production and swallow dysfunction in Parkinson’s disease. Dysphagia. 2008;23(3):297–301. Epub 2008/05/17. doi: 10.1007/s00455-007-9144-x ; PubMed Central PMCID: PMC4080193. 18483823 PMC4080193

[69] Pitts T, Troche M, Mann G, Rosenbek J, Okun MS, Sapienza C, et al. Using voluntary cough to detect penetration and aspiration during oropharyngeal swallowing in patients with Parkinson disease. CHEST. 2010;138(6):1426–31. doi: 10.1378/chest.10-0342 . Language: English. Entry Date: 20705802. Revision Date: 20200708. Publication Type: journal article. 20705802

[70] Troche MS, Brandimore AE, Okun MS, Davenport PW, Hegland KW. Decreased cough sensitivity and aspiration in Parkinson disease. Chest. 2014;146(5):1294–9. Epub 2014/06/27. doi: 10.1378/chest.14-0066 ; PubMed Central PMCID: PMC4219343. 24968148 PMC4219343

[71] Trebbia G, Lacombe M, Fermanian C, Falaize L, Lejaille M, Louis A, et al. Cough determinants in patients with neuromuscular disease. Respiratory physiology & neurobiology. 2005;146(2–3):291–300. doi: 10.1016/j.resp.2005.01.001 15766917

[72] Chiara T, Martin AD, Davenport PW, Bolser DC. Expiratory Muscle Strength Training in Persons with Multiple Sclerosis Having Mild to Moderate Disability: Effect on Maximal Expiratory Pressure, Pulmonary Function, and Maximal Voluntary Cough. Archives of Physical Medicine and Rehabilitation 2006;87(4):468–73. doi: 10.1016/j.apmr.2005.12.035 16571384 PMC3121162

[73] Tawara Y, Fujishima I, Katagiri N, Arizono S, Ohgi S, Kozu R. Effect of expiratory muscle strength training on cough and swallowing in patients with dysphagia following stroke. European Respiratory Journal. 2018;52(suppl 62):PA1452. doi: 10.1183/13993003.congress-2018.PA1452

[74] Kim J, Davenport P, Sapienza C. Effect of expiratory muscle strength training on elderly cough function. Archives of Gerontology and Geriatrics. 2009;48(3):361–6. doi: 10.1016/j.archger.2008.03.006 18457885

[75] Jo M-R, Kim N-S. The correlation of respiratory muscle strength and cough capacity in stroke patients. J Phys Ther Sci. 2016;28(10):2803–5. Epub 2016/10/28. doi: 10.1589/jpts.28.2803 . 27821939 PMC5088130

[76] Park JH, Kang S-W, Lee SC, Choi WA, Kim DH. How respiratory muscle strength correlates with cough capacity in patients with respiratory muscle weakness. Yonsei medical journal. 2010;51(3):392–7. doi: 10.3349/ymj.2010.51.3.392 . 20376892 PMC2852795

[77] Chaitra B, Vijay M. Effect of Aerobic Exercise Training on Peak expiratory Flow Rate: A Pragmatic Randomized Controlled Trial. Int J Biol Med Res 2011;2(3):789–92.

[78] Bassi R, Sharma S, Sharma A, Kaur D, Kaur H. The effect of aerobic exercises on peak expiratory flow rate and physical fitness index in female subjects. 2015;5(5):376–81.

[79] Farid R, Farahzad JA, Atri AE, Rahimi MB, Khaledan A, Talaei-Khoei M, et al. Effect of Aerobic Exercise Training on Pulmonary Function and Tolerance of Activity in Asthmatic Patients. Iranian Journal of Allergy, Asthma, and Immunology. 2005;4(3):133–8. 17301436

[80] Hansen EF, Vestbo J, Phanareth K, Kok-Jensen A, Dirksen A. Peak flow as predictor of overall mortality in asthma and chronic obstructive pulmonary disease. Am J Respir Crit Care Med. 2001;163(3 Pt 1):690–3. Epub 2001/03/20. doi: 10.1164/ajrccm.163.3.2006120 . 11254525

[81] Quanjer PH LM, Gregg I, Miller MR, Pedersen OF. Peak expiratory flow: conclusions and recommendations of a Working Party of the European Respiratory Society. Eur Respir J. 1997;10:2s–8s. 9098701

[82] Silveira RA, Trippo K.V, Duarte GP, Neto G.N., Filho JO, Ferraz DD. The Effects of Functional Training and Stationary Cycling on Respiratory Function of Elderly with Parkinson Disease: A Pilot Study. Fisioterapia em Movimento. 2018;21.

[83] Sapienza CM, Wheeler K. Respiratory muscle strength training: Functional outcomes versus plasticity. Seminars in Speech and Language. 2006;27(4):236–44. doi: 10.1055/s-2006-955114 17117350

[84] Sharma NK, Robbins K, Wagner K, Colgrove YM. A randomized controlled pilot study of the therapeutic effects of yoga in people with Parkinson’s disease. Int J Yoga. 2015;8(1):74–9. Epub 2015/01/06. doi: 10.4103/0973-6131.146070 ; PubMed Central PMCID: PMC4278140. 25558138 PMC4278140

[85] Main E, Denehy L, Webber BA, Pryor JA, Ammani Prasad S. Cardiorespiratory physiotherapy: adults and paediatrics. Fifth ed. Edinburgh: Elsevier; 2016.

[86] Rodríguez-Molinero A, Narvaiza L, Ruiz J, Gálvez-Barrón C. Normal respiratory rate and peripheral blood oxygen saturation in the elderly population. J Am Geriatr Soc. 2013;61(12):2238–40. Epub 2013/12/18. doi: 10.1111/jgs.12580 . 24329828

[87] Aquino YC, Cabral LM, Miranda NC, Naccarato MC, Falquetto B, Moreira TS, et al. Respiratory disorders of Parkinson’s disease. Journal of Neurophysiology. 2022;127(1):1–15. doi: 10.1152/jn.00363.2021 . 34817281

[88] Kaczynska K, Orlowska ME, Andrzejewski K. Respiratory Abnormalities in Parkinson’s Disease: What Do We Know from Studies in Humans and Animal Models? Int J Mol Sci. 2022;23(7). Epub 2022/04/13. doi: 10.3390/ijms23073499 ; PubMed Central PMCID: PMC8998219. 35408858 PMC8998219

[89] Åstrand P-O, Rodahl K. Textbook of work physiology: physiological bases of exercise. 2nd ed. ed: New York (N.Y.): McGraw-Hill; 1977.

[90] Selvamurugamani S, Senthilkumar P. Efficacy of cardio resistance training and concurrent training on resting metabolic rate and respiratory rate among sedentary males. 2019.

[91] Mavrommati F, Collett J, Franssen M, Meaney A, Sexton C, Dennis-West A, et al. Exercise response in Parkinson’s disease: insights from a cross-sectional comparison with sedentary controls and a per-protocol analysis of a randomised controlled trial. BMJ Open. 2017;7(12):e017194. Epub 2017/12/29. doi: 10.1136/bmjopen-2017-017194 ; PubMed Central PMCID: PMC5770916. 29282259 PMC5770916

[92] Crowley E, Powell C, Carson BP, W. Davies R. The Effect of Exercise Training Intensity on VO2max in Healthy Adults: An Overview of Systematic Reviews and Meta-Analyses. Translational Sports Medicine. 2022;2022:9310710. doi: 10.1155/2022/9310710 PMC11022784 38655159

[93] Stanley RK, Protas EJ, Jankovic J. Exercise performance in those having Parkinson’s disease and healthy normals. Med Sci Sports Exerc. 1999;31(6):761–6. doi: 10.1097/00005768-199906000-00001 . 10378900

[94] Thrue C, Hvid LG, Gamborg M, Dawes H, Dalgas U, Langeskov-Christensen M. Aerobic capacity in persons with Parkinson’s disease: a systematic review. Disabil Rehabil. 2022:1–13. Epub 2022/07/12. doi: 10.1080/09638288.2022.2094480 . 35815568

[95] Gjellesvik TI, Becker F, Tjønna AE, Indredavik B, Lundgaard E, Solbakken H, et al. Effects of High-Intensity Interval Training After Stroke (The HIIT Stroke Study) on Physical and Cognitive Function: A Multicenter Randomized Controlled Trial. Archives of Physical Medicine and Rehabilitation.2021;102(9):1683–91. doi: 10.1016/j.apmr.2021.05.008 34102144

[96] Neuendorf T, Haase R, Schroeder S, Schumann M, Nitzsche N. Effects of high-intensity interval training on functional performance and maximal oxygen uptake in comparison with moderate intensity continuous training in cancer patients: a systematic review and meta-analysis. Support Care Cancer. 2023;31(12):643. Epub 2023/10/18. doi: 10.1007/s00520-023-08103-9 ; PubMed Central PMCID: PMC10584719. 37851104 PMC10584719

[97] Ferté J-B, Boyer FC, Taiar R, Pineau C, Barbe C, Rapin A. Impact of resistance training on the 6-minute walk test in individuals with chronic obstructive pulmonary disease: A systematic review and meta-analysis. Annals of Physical and Rehabilitation Medicine. 2022;65(3):101582. doi: 10.1016/j.rehab.2021.101582 34626862

[98] Wehner C, Blank C, Arvandi M, Wehner C, Schobersberger W. Effect of Tai Chi on muscle strength, physical endurance, postural balance and flexibility: a systematic review and meta-analysis. BMJ Open Sport Exerc Med. 2021;7(1):e000817. Epub 2021/02/23. doi: 10.1136/bmjsem-2020-000817 ; PubMed Central PMCID: PMC7871341. 33614126 PMC7871341

[99] Canning CG, Allen NE, Dean CM, Goh L, Fung VS. Home-based treadmill training for individuals with Parkinson’s disease: a randomized controlled pilot trial. Clinical Rehabilitation. 2012;26(9):817–26. doi: 10.1177/0269215511432652 . 22257506

[100] Zhen K, Zhang S, Tao X, Li G, Lv Y, Yu L. A systematic review and meta-analysis on effects of aerobic exercise in people with Parkinson’s disease. npj Parkinson’s Disease. 2022;8(1):146. doi: 10.1038/s41531-022-00418-4 36316416 PMC9622812

[101] Duncan RP, Earhart GM. Randomized Controlled Trial of Community-Based Dancing to Modify Disease Progression in Parkinson Disease. Neurorehabilitation and Neural Repair. 2011;26(2):132–43. doi: 10.1177/1545968311421614 21959675

[102] Gamborg M, Hvid LG, Dalgas U, Langeskov‐Christensen M. Parkinson’s disease and intensive exercise therapy—An updated systematic review and meta‐analysis. Acta Neurologica Scandinavica. 2022;145(5):504–28. doi: 10.1111/ane.13579 34997759

[103] Sabino-Carvalho JL, Vianna LC. Altered cardiorespiratory regulation during exercise in patients with Parkinson’s disease: A challenging non-motor feature. SAGE Open Medicine. 2020;8:205031212092160. doi: 10.1177/2050312120921603 32435491 PMC7222646

[104] Ellis TD, Colón-Semenza C, DeAngelis TR, Thomas CA, Hilaire MS, Earhart GM, et al. Evidence for Early and Regular Physical Therapy and Exercise in Parkinson’s Disease. Semin Neurol. 2021;41(2):189–205. Epub 2021/03/21. doi: 10.1055/s-0041-1725133 ; PubMed Central PMCID: PMC8678920. 33742432 PMC8678920

[105] Hortobágyi T, Sipos D, Borbély G, Áfra G, Reichardt-Varga E, Sántha G, et al. Detraining Slows and Maintenance Training Over 6 Years Halts Parkinsonian Symptoms-Progression. Frontiers in Neurology. 2021;12. doi: 10.3389/fneur.2021.737726 34867721 PMC8641297

[106] Bonanni R, Cariati I, Tarantino U, D’Arcangelo G, Tancredi V. Physical Exercise and Health: A Focus on Its Protective Role in Neurodegenerative Diseases. J Funct Morphol Kinesiol. 2022;7(2). Epub 2022/06/02. doi: 10.3390/jfmk7020038 ; PubMed Central PMCID: PMC9149968. 35645300 PMC9149968

[107] Oliveira de Carvalho A, Filho ASS, Murillo-Rodriguez E, Rocha NB, Carta MG, Machado S. Physical Exercise For Parkinson’s Disease: Clinical And Experimental Evidence. Clin Pract Epidemiol Ment Health. 2018;14:89–98. Epub 2018/05/23. doi: 10.2174/1745017901814010089 ; PubMed Central PMCID: PMC5897963. 29785199 PMC5897963

[108] Carter JH, Nutt JG, Woodward WR. The effect of exercise on levodopa absorption. Neurology. 1992;42(10):2042–. doi: 10.1212/wnl.42.10.2042 1407589

[109] Muhlack S, Welnic J, Woitalla D, Müller T. Exercise improves efficacy of levodopa in patients with Parkinson’s disease. Mov Disord. 2007;22(3):427–30. Epub 2007/01/18. doi: 10.1002/mds.21346 . 17226855

[110] Reuter I, Harder S, Engelhardt M, Baas H. The effect of exercise on pharmacokinetics and pharmacodynamics of levodopa. Movement Disorders. 2000;15(5):862–8. doi: 10.1002/1531-8257(200009)15:5<862::aid-mds1015>3.0.co;2-s 11009191

