# Supplementary material for: Responsiveness of respiratory function in Parkinson’s Disease to an integrative exercise programme: A prospective cohort study
Source: PLoS One. 2024 Mar 29;19(3):e0301433. doi: 10.1371/journal.pone.0301433 (PMC10980210; doi:10.1371/journal.pone.0301433)
Supplement: S1 Checklist — (DOCX) [file pone.0301433.s001.docx]

**STROBE Statement**

**Responsiveness of respiratory function in Parkinson’s Disease to an integrative exercise programme: A prospective cohort study.**

|  | | Item No. | Recommendation | Page  No. | Relevant text from manuscript |
| --- | --- | --- | --- | --- | --- |
| **Title and abstract** | | | | | |
| Title and abstract | | 1 | (*a*) Indicate the study’s design with a commonly used term in the title or the abstract | 1 | Responsiveness of Respiratory Function in Parkinson’s Disease to an Integrative Exercise Programme: A Prospective Cohort Study |
|  |  |  | (*b*) Provide in the abstract an informative and balanced summary of what was done and what was found | 2 | Twenty-three people with PD (median H&Y=2) self-selected to participate in this exploratory prospective cohort study. Evaluation of participants occurred at three time points: at baseline; following the 12-week exercise programme and at 4-month follow-up.  Compared to published norms, participants had lower inspiratory (p<0.001) and expiratory muscle strength (p<0.001), PCF (p=0.002), 6MWT (p<0.001), FEV (p<0.04), FVC (p<0.02), and PEF (p<0.001). MEP and 6MWT increased significantly from baseline to post intervention (MD: 13.8, 95% CI: 2.5, 25.1, p=0.013 and MD: 74.4, 95% CI: 40.6, 108.2 p<0.001 respectively) but gains were not maintained at 4-month follow-up. Only PEF was noted to improve post intervention with gains maintained at 4 months (MD: 50.2, 95% CI: 2.8, 97.5, p=0.04). |
| Introduction | | | | |  |
| Background/rationale | | 2 | Explain the scientific background and rationale for the investigation being reported | 5 | This nested study was conducted to better understand common respiratory dysfunction in people with PD presenting to a community-based exercise programme and to identify measures of dysfunction that are potentially responsive to the integrative exercise offering delivered in a university-setting |
| Objectives | | 3 | State specific objectives, including any prespecified hypotheses | 5 | Baseline respiratory measures capturing restrictive, obstructive, cough dysfunction, respiratory muscle strength and cardiovascular fitness were established and compared with matched, published population norms. Immediate and longer-term effects of the 12-week mixed exercise programme on sub-optimal respiratory measures were examined in participants with Parkinson’s Disease. |
| Methods | | | | |  |
| Study design | | 4 | Present key elements of study design early in the paper | 6 | Study Design: An exploratory prospective cohort study. |
| Setting | | 5 | Describe the setting, locations, and relevant dates, including periods of recruitment, exposure, follow-up, and data collection | 6 | Data collection took place in a laboratory setting and the exercise programme was delivered as part of a community initiative delivered at a university gym setting.  Evaluation of participants occurred at three time points during the study, an initial baseline assessment, following completion of the 12-week programme and at 16-weeks post programme completion. |
| Participants | | 6 | (*a*) *Cohort study*—Give the eligibility criteria, and the sources and methods of selection of participants. Describe methods of follow-up  *Case-control study*—Give the eligibility criteria, and the sources and methods of case ascertainment and control selection. Give the rationale for the choice of cases and controls  *Cross-sectional study*—Give the eligibility criteria, and the sources and methods of selection of participants | 6 | Members of a local PD organisation were invited to participate in the exercise programme at the university prior to commencement of the study. An information leaflet was provided to all potential participants. Participants were required to have a confirmed diagnosis of Parkinson’s Disease and be under the care of a consultant neurologist. All study participants, volunteers by self-selection, provided written informed consent prior to participation.  Exclusion criteria: chronic respiratory disease diagnosis (e.g., COPD), acute respiratory tract infection, unstable medical conditions (e.g. uncontrolled diabetes), and dementia. |
|  |  |  | (*b*) *Cohort study*—For matched studies, give matching criteria and number of exposed and unexposed  *Case-control study*—For matched studies, give matching criteria and the number of controls per case | n/a | n/a |
| Variables | | 7 | Clearly define all outcomes, exposures, predictors, potential confounders, and effect modifiers. Give diagnostic criteria, if applicable | 7+8 | Basic demographic details and history of PD and respiratory illnesses were recorded using a proforma. Anthropometric measurements were taken. Participants were assessed using the MDS-UPDRS [28] and total and subscale scores calculated [29]. Medication on or off phase was recorded for those on levodopa medication. Respiratory rate was calculated with participants sitting in a chair, the number of breaths in a 60 second period was recorded while oxygen saturation was being recorded.  Spirometry Assessment: Lung volume (Forced Vital Capacity) and flow rates (Forced Expiratory Volume in 1 second, ratio of Forced Expiratory Volume in 1 second to Forced Vital Capacity (FEV1/FVC) and Peak Expiratory Flow (PEF) were measured using a Spirometer (Micro1; Carefusion). The assessment protocol followed the ATS/ERS task force standardisation of lung function testing guidelines[30]. Normative values were sourced from the ERS task force GLI predicted values [31] for FVC, FEV1 and FEV1/FVC. The NAHNES normal values [32] were used for PEF.  Respiratory Muscle Strength Assessment: Inspiratory Muscle Strength (MIP), Expiratory Muscle Strength (MEP) and Sniff nasal inspiratory pressure (SNIP) were measured using a pressure transducer (MicroRPM; Carefusion) and standardised using the ATS/ERS Statement on Respiratory Muscle Testing. For comparison with normative data, male and female normative values for MIP, MEP and SNIP were obtained from Uldry et al, 1995 [33].  Cough Assessment: Peak Cough Flow (PCF), a measure of gross respiratory function (strength, flow rate, and respiratory hygiene composite measure) was measured using a PCF meter (Vitalograph & facemask). As there is no standardised testing guideline for PCF, the test was standardised by having participants in standing with the facemask placed over their nose and mouth by the investigator and instructed to “take a breath in and then cough as strongly as you can” following a demonstration by the examiner. The best of 3 attempts was documented as the PCF. For comparison with normative data, a PCF of 360+l/min was considered to be an effective cough, in line with the published literature [34, 35]  Cardiovascular Fitness Assessment: VO2 max was estimated using the YMCA submaximal cycle ergometry test protocol [36], following the ACSM guidelines [37]. Participants sat on an upright cycling ergometer and pedalled for 3 minutes at zero resistance as a warm-up. Initial work rate was then set at 50 watts for 3 minutes and increased 3 minutes later (minute 6 of the testing protocol) to 70W. Participants pedalled at this resistance for a further 3 minutes, followed by a 3-minute cool down pedalling period. HR, BP and SaO2 were recorded at each 3-minute time point during testing. VO2 max was estimated from the regression line generated between work rate and heart rate responses at each stage. The maximum HR (220-age) was used to estimate what the maximal work rate would be. This work rate was converted to VO2 max (estimated) using the ACSM equation (VO2 (ml.kg-1.min-1) = 7 + (1.8 x work rate) / body mass (wt. in kg)) for cycle ergometry. Normative reference range for non-athletic males and females by age group were used as the comparator value [38], with the higher value applied. The Six Minute Walk Test (6MWT) was employed to assess functional cardiovascular fitness. This was standardised following the American Thoracic Society (ATS) guidelines for the six-minute walk test [39]. The following predictive equations derived age and gender matched normative values: males: 6MWT (m) = 867 - (5.71 x age in years) +(1 .03 x height in cm), females: 6MWT (m) = 525 - (2.86 x age in years) + (0.71 x height in cm) – (6.22 x BMI) [40]. |
| Data sources/ measurement | | 8* | For each variable of interest, give sources of data and details of methods of assessment (measurement). Describe comparability of assessment methods if there is more than one group | *a/a* | *a/a* |
| Bias | | 9 | Describe any efforts to address potential sources of bias | n/a | n/a |
| Study size | | 10 | Explain how the study size was arrived at | 19 | One acknowledged limitation in the study is the small sample size which means the study may not be adequately powered to detect statistically significant change in the values which did improve over the programme of exercise. Effect sizes for all changes in measures observed were medium or large showing promise as preliminary findings that now warrant testing in a larger, powered study with a control group. |
| Quantitative variables | | 11 | Explain how quantitative variables were handled in the analyses. If applicable, describe which groupings were chosen and why | 9 | Descriptive statistics (mean (SD), median (range) and n (%)) summarised baseline data. Independent t-tests explored differences in baseline respiratory measures against normative data for parametric data and Mann Whitney U tests for non-parametric data |
| Statistical methods | | 12 | (*a*) Describe all statistical methods, including those used to control for confounding | 9-10 | Descriptive statistics (mean (SD), median (range) and n (%)) summarised baseline data. Independent t-tests explored differences in baseline respiratory measures against normative data for parametric data and Mann Whitney U tests for non-parametric data. A repeated measures ANOVA was performed to examine the difference in measures over the three time points, pre-intervention, post intervention and at follow up. Post hoc analysis, with Bonferroni correction, examined changes between the different time points. Where results indicate a large variation in class attendances, further analysis examining the effects of attendance rates will be conducted. |
|  |  |  | (*b*) Describe any methods used to examine subgroups and interactions | 9-10 | A mixed ANOVA examined whether change in respiratory measures over time was affected by H&Y stage. Where no effect for H&Y stage was observed, the ANOVA was rerun reporting change overtime without H&Y. |
|  |  |  | (*c*) Explain how missing data were addressed | 13 | The 2 participants who were excluded from the spin classes due to abnormal haemodynamic response to exercise identified during pre-screening were not considered in attendance numbers. |
|  |  |  | (*d*) *Cohort study*—If applicable, explain how loss to follow-up was addressed  *Case-control study*—If applicable, explain how matching of cases and controls was addressed  *Cross-sectional study*—If applicable, describe analytical methods taking account of sampling strategy | n/a | n/a |
|  |  |  | (*e*) Describe any sensitivity analyses | n/a | n/a |
| Results | | | | | |
| Participants | | 13* | (a) Report numbers of individuals at each stage of study—eg numbers potentially eligible, examined for eligibility, confirmed eligible, included in the study, completing follow-up, and analysed | 10 | Twenty-three volunteers by self-selection participated in this study. No participant dropped out of the exercise programme; two participants were excluded from the spinning classes due to abnormal haemodynamic responses to aerobic exercise identified during pre-screening. |
|  |  |  | (b) Give reasons for non-participation at each stage | 10 | No participant dropped out of the exercise programme; two participants were excluded from the spinning classes due to abnormal haemodynamic responses to aerobic exercise identified during pre-screening. |
|  |  |  | (c) Consider use of a flow diagram | 10 | Table 2  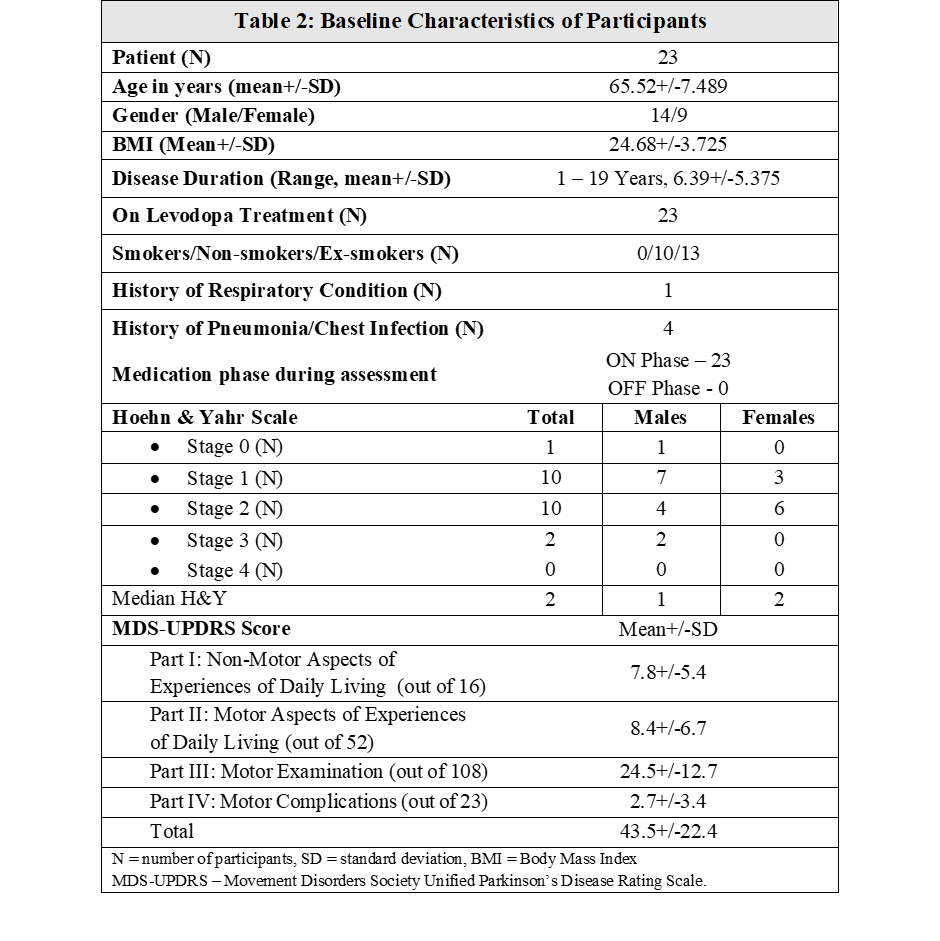 |
| Descriptive data | | 14* | (a) Give characteristics of study participants (eg demographic, clinical, social) and information on exposures and potential confounders | 10 | See Table 2 |
|  |  |  | (b) Indicate number of participants with missing data for each variable of interest | 10 | See table 2 |
|  |  |  | (c) *Cohort study*—Summarise follow-up time (eg, average and total amount) | n/a | n/a |
| Outcome data | | 15* | *Cohort study*—Report numbers of outcome events or summary measures over time | *12, 13* | *See table 3 & 4* |
|  |  |  | *Case-control study—*Report numbers in each exposure category, or summary measures of exposure | *n/a* | *n/a* |
|  |  |  | *Cross-sectional study—*Report numbers of outcome events or summary measures | *n/a* | *n/a* |
| Main results | | 16 | (*a*) Give unadjusted estimates and, if applicable, confounder-adjusted estimates and their precision (eg, 95% confidence interval). Make clear which confounders were adjusted for and why they were included | *12, 13* | *See table 3 & 4* |
|  |  |  | (*b*) Report category boundaries when continuous variables were categorized | n/a | n/a |
|  |  |  | (*c*) If relevant, consider translating estimates of relative risk into absolute risk for a meaningful time period | n/a | n/a |
| Other analyses | | 17 | Report other analyses done—eg analyses of subgroups and interactions, and sensitivity analyses | n/a | n/a |
| Discussion | | | | | |
| Key results | | 18 | Summarise key results with reference to study objectives | 15 | This study, which tested an opt-in, community based integrative exercise programme comprising three different types of exercise classes in PD, demonstrated statistically significant reductions in MDS-UPDRS Scores over the 12 weeks, that were sustained at follow-up. This was not an unexpected finding given evidence from both human and animal models that exercise has a neuroprotective role and can improve motor impairments and physical condition in people with PD regardless of disease staging. Measuring respiratory function in PD is not standard practice despite evidence of obstructive, restrictive, mixed, and central issues reported in the literature [13]. Similarly, inclusion of outcomes addressing the spectrum of respiratory dysfunction is not routine in exercise trials in PD, with limited evidence to support their efficacy to affect improvements in respiratory function [26]. This study identified significant baseline impairments in respiratory muscle strength (inspiratory and expiratory), PCF rates and cardiovascular fitness (6MWT) and abnormalities in spirometry measures addressing restrictive (FVC) and obstructive (PEF) dysfunction, in participants presenting to a community-based exercise class for PD. These are interesting findings in the context of the study population where the majority are in the early stages of disease progression (87% in H&Y stages 1-2), although preclinical studies have previously highlighted early respiratory impairments in PD [45],[46]. The 12-week opt-in integrative exercise programme targeting resistance training and balance (functional circuit training classes and tai chi) and aerobic training (spin classes) affected short term, statistically significant improvements in expiratory muscle strength, PEF and the 6MWT. Non-significant improvements were noted in inspiratory muscle strength and estimated Vo2max. Improvements were not maintained long term (except for PEF rate), suggesting ongoing input is required for maintenance. Resting respiratory rate remained largely unchanged and PCF rate was noted to decline over the testing period. Elements of respiratory dysfunction identified in this study appear to be responsive to exercise in people with PD in the short term. With high attendance at all class types, analysis of responsiveness by exercise type was not possible. |
| Limitations | | 19 | Discuss limitations of the study, taking into account sources of potential bias or imprecision. Discuss both direction and magnitude of any potential bias | 19 | One acknowledged limitation in the study is the small sample size which means the study may not be adequately powered to detect statistically significant change in the values which did improve over the programme of exercise. |
| Interpretation | | 20 | Give a cautious overall interpretation of results considering objectives, limitations, multiplicity of analyses, results from similar studies, and other relevant evidence | 19-20 | This study explored how an integrative, non-specific exercise programme could identify impairments and enhance respiratory function in PD. This differs from previous studies that employed targeted, clinical intervention/s that could be considered less ecologically valid in comparison to community-based exercise programmes, available to large groups of people outside of a medical setting. While the intervention tested in this study allowed participants freedom of choice in their exercise program, all participants attended a high proportion of all classes. It was not possible to examine further which exercise component/s may have offered greatest benefit to the respiratory metrics which improved post-intervention, as previous research identifies specificity of exercise is required to target respiratory dysfunction [100]. Therefore, to ensure greater numbers of people with PD have access to effective exercise supports, future work is needed to determine which type, or combination of types of community-based exercise regimes offer the greatest improvement in respiratory function and greatest all-round benefits i.e., cardiovascular fitness, respiratory function, mobility; strength/balance/coordination, enjoyment, social interaction and support, cognitive function. |
| Generalisability | | 21 | Discuss the generalisability (external validity) of the study results | 19 | Effect sizes for all changes in measures observed were medium or large showing promise as preliminary findings that now warrant testing in a larger, powered study with a control group. In addition, most participants in this study had mild PD symptoms (H&Y 0-2) therefore the findings presented at baseline and in response to exercise may not be representative of all PD patients. |
| Other information |  | | | | |
| Funding | | 22 | Give the source of funding and the role of the funders for the present study and, if applicable, for the original study on which the present article is based | n/a | n/a |
